# Exploring Workarounds Related to Electronic Health Record System Usage: A Study Protocol

**DOI:** 10.2196/resprot.6766

**Published:** 2017-04-28

**Authors:** Vincent Blijleven, Kitty Koelemeijer, Monique Jaspers

**Affiliations:** ^1^ Center for Marketing & Supply Chain Management Nyenrode Business University Breukelen Netherlands; ^2^ Academisch Medisch Centrum Department of Medical Informatics University of Amsterdam Amsterdam Netherlands

**Keywords:** electronic health records, qualitative research, workarounds, unintended consequences, physicians, nurses, administrative personnel

## Abstract

**Background:**

Health care providers resort to informal temporary practices known as workarounds for handling exceptions to normal workflow that are unintentionally imposed by electronic health record (EHR) systems. Although workarounds may seem favorable at first sight, they are generally suboptimal and may jeopardize patient safety, effectiveness, and efficiency of care. Identifying workarounds and understanding their motivations, scope, and impact is pivotal to support the design of user-friendly EHRs and achieve closer alignment between EHRs and work contexts.

**Objective:**

We propose a study protocol to identify EHR workarounds and subsequently determine their scope and impact on health care providers’ workflows, patient safety, effectiveness, and efficiency of care. First, knowing whether a workaround solely affects the health care provider who devised it, or whether its effects extends beyond the EHR user to the work context of other health care providers, is key to accurately assessing its degree of influence on the overall patient care workflow. Second, knowing whether the consequence of an EHR workaround is favorable or unfavorable provides insights into how to address EHR-related safety, effectiveness, and efficiency concerns. Knowledge of both perspectives can provide input on optimizing EHR designs.

**Methods:**

In the study, a combination of direct observations, semistructured interviews, and qualitative coding techniques will be used to identify, analyze, and classify EHR workarounds. The research project will be conducted within three distinct pediatric care processes and settings at a large university hospital.

**Results:**

Data was collected using the described approach from January 2016 to March 2017. Data analysis is underway and is expected to be completed in May 2017. We aim to report the results of this study in a follow-up publication.

**Conclusions:**

This study protocol provides a grounded framework to explore EHR workarounds from a holistic and integral perspective. Insights from this study can inform the design and redesign of EHRs to further align with work contexts of healthcare professionals, and subsequently lead to better organization and safer provision of care.

## Introduction

In recent years, an increasing number of hospitals around the world have implemented electronic health record (EHR) systems [1-3]. According to 2015 American Hospital Association survey data, 83.8% of all US non-federal acute care hospitals have adopted at least a basic EHR, representing an almost nine-fold increase since 2008 (9.4%) [4]. EHRs can improve the ways that medical information is stored, communicated, and processed by those involved in delivering health care [5]. Preventable adverse events in health care frequently relate to the unavailability of important patient information, but in this context health information technology such as EHRs hold promise for improving the quality of information transfer [6].

Multiple studies have reported on desirable outcomes of EHRs. Examples include improvements related to patient safety [7-9], quality of care [9-11], efficiency [9,12-15], and reduced costs [16,17]. However, achieving these merits expected from EHRs is far from evident. Many other studies likewise address unfavorable and often unanticipated outcomes of adopting EHRs, such as health care providers suffering from *alert fatigue* [18,19], paper persistence [20-22], workflow mismatches [23], difficulties in finding the right information in the systems [24], never-ending system demands [25], or an EHR interface that is unsuitable for a highly interruptive health care context [26].

These undesirable and unanticipated consequences of EHR adoption can have negative and unintended effects on the overall health care organization and its work processes (and the outcomes thereof), and have frequently been subject to further examination. When the practices of health care providers are unintentionally but negatively influenced by mismatches between EHR designs and actual workflows, providers devise so-called *workarounds*. Workarounds can be defined as, “informal temporary practices for handling exceptions to normal workflow” [27] or, “staff actions that do not follow explicit or implicit rules, assumptions, workflow regulations, or intentions of systems designers” [28]. Workarounds are solutions to workflow mismatches that help to coordinate work, especially under conditions of high time pressure. Existing literature shows that system users may devise workarounds as a consequence of: a perceived lack of efficiency causing the user to execute the task at hand in a different manner, task complexity dictating workflow or system functionality issues, no correct or desired option being available in the system-dictated workflow, no options for customizing the system output, or a lack of trust in electronic versus paper-based communication [20-22,26,27,29].

Although workarounds may solve the exceptions that EHRs impose upon the ordinary workflows of their users, they are generally suboptimal, as the EHR fails to live up to the goals of its implementation (ie, improving the practices of health care providers) and may negatively influence the safety, effectiveness, and efficiency of care. Understanding why and how workarounds occur is pivotal to develop user-friendly EHRs, and to achieve greater alignment between work context and the EHR [22]. Research into workarounds has a prominent place in health care, and workarounds have been identified, analyzed, and described in various contexts [20,30-34].

To date, research into the scope and impact of EHR usage-related workarounds on overall patient care processes has been limited. First, concerning the scope of EHR workarounds, it is crucial to know whether a workaround affects a single EHR user who devised it, or whether its effects extend beyond the EHR user to the work context of other health care providers, to accurately assess its impact on the overall patient care workflow. Second, knowing whether the consequence of an EHR workaround is favorable or unfavorable provides insights into how to address EHR-related safety, effectiveness, and efficiency concerns. Knowledge of both perspectives can provide input on optimizing EHR design.

This study protocol proposes a way of identifying, analyzing, and classifying EHR workarounds to determine their scope and impact on the patient care process. Within a large university hospital, we intend to conduct direct observations of (and semistructured interviews with) health care providers while they use EHRs in three different processes, each taking place in a distinct physical environment: the preparation of outpatient consultations in private offices of health care professionals, actual outpatient consultations in examination rooms, and actual inpatient consultations with admitted patients in wards. The research design, clinical setting, and methods to be used in the research project are described in the following section.

## Methods

### Study Design

**Figure 1:**
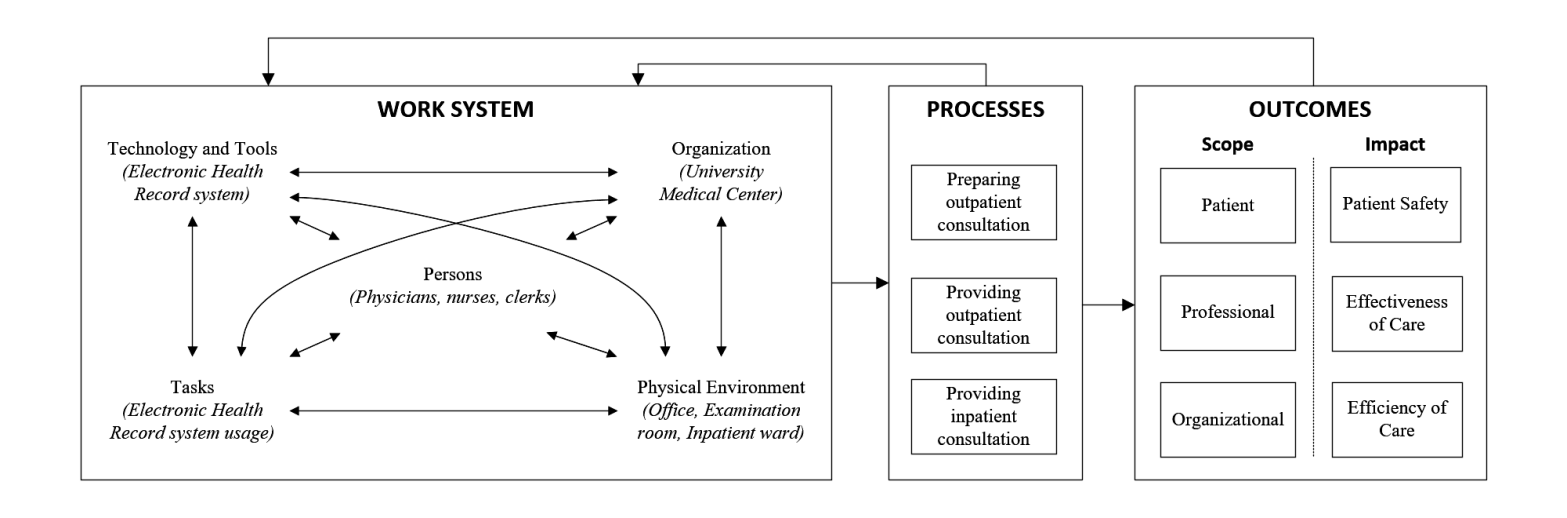
Conceptual framework to study electronic health record workarounds, adapted from Holden et al [35].

To address the aim of determining the scope and impact of EHR-related workarounds, we adapted one of the most widely used health care human factors systems frameworks, the Systems Engineering Initiative for Patient Safety (SEIPS) framework [35]. The SEIPS framework corresponds with Donabedian’s structure-process-outcome framework [36] for examining health care services and evaluating quality of health care. SEIPS provides an integral conceptual framework for applying systems engineering concepts to identify and analyze workarounds in specific health care contexts. Workarounds in health care settings have been found to differ as a function of people’s roles, and can have a cascading effect (meaning that workarounds can trigger a series of further workarounds) [27]. Using the integrated and holistic perspective of the SEIPS framework, relationships between health care work systems, processes, and resulting outcomes can be studied together, rather than each in isolation. The SEIPS framework has already proven valuable in studying workarounds in various health care contexts [28,37]. As illustrated in Figure 1, the framework consists of three main components: the *work system,* including persons, tasks, tools and technologies, organization, and the internal environment; *processes* within the work system; and *outcomes* that result from those processes.

In our study *technology* concerns the EHR that is used by physicians, nurses, and clerks (ie, the *persons*) within a university hospital (ie, the *health care organization*) located in the Netherlands. The university hospital adopted the EHR in 2015. Over 8000 hospital staff work with the EHR and all medication, blood, laboratory, and x-rays tests are ordered through the EHR. Before the university hospital implemented this EHR, a central hospital information system interfaced with multiple ancillary systems, including: a Computerized Physician Order Entry (CPOE) system for ordering medication and laboratory tests, a hospital pharmacy information system, and a hospital-wide scheduling system.

In the study, we will investigate workarounds by means of direct observations and semistructured interviews in three processes. Each process will take place in a distinct physical environment: the preparation of outpatient consultations in private offices of health care professionals, actual outpatient consultation in examination rooms, and actual inpatient consultation with patients admitted into wards. Workarounds occurring within these three processes can have consequences that affect the outcomes of each process. We will determine the scope of each workaround to the patient, the health care professional, and the overall organization level, or a combination thereof. Furthermore, to determine the impact of each workaround, we will classify whether its consequence is favorable or unfavorable, and assess its impact on patient safety, patient care effectiveness, and efficiency.

**Figure 2:**
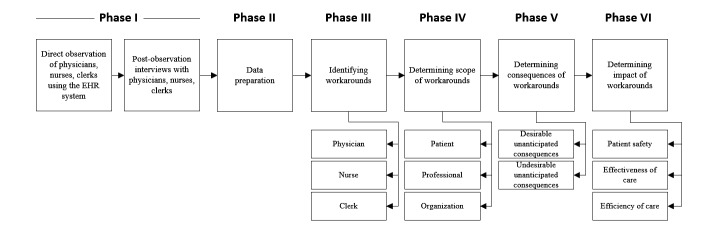
Illustration of the six research phases to be conducted. EHR: electronic health record.

Due to the unique nature of each health care setting to be studied, the direct observations and semistructured interview procedures will vary per setting. The research project involves six major chronological phases, as illustrated in Figure 2. The following subsections address the proposed research methods and practical execution for each phase in greater detail. A summary of the data collection and analysis plans for all three settings is provided in Table 1.

**Table 1:** Summary of research design by process to be studied.

Process	Preparing outpatient consultation	Providing outpatient consultation	Providing inpatient consultation
**Sample**	Approximately 12 physicians, 6 nurses, and 3 clerks (same staff as in *providing outpatient consultation* process)	Approximately 12 physicians, 6 nurses, and 3 clerks (same staff as in *preparing outpatient consultation* process)	Approximately 12 physicians, 6 nurses, and 3 clerks
**Participant selection criteria**	(1) Must have completed the required training to use EHR, and (2) must have used EHR from the moment of its implementation	(1) Must have completed the required training to use EHR, and (2) must have used EHR from the moment of its implementation	(1) Must have completed the required training to use EHR, and (2) must have used EHR from the moment of its implementation
**Setting**	Private office	Examination room	Inpatient ward
**Interaction**	User-system	User-patient, user-system	User-patient, user-system
**Procedure (per person)**	Direct observation while preparing outpatient consultation (1-2 hours), asking opportunistic questions while observing, semistructured follow-up interviews (1 hour)	Direct observation while providing outpatient consultation (4-6 hours), semistructured follow-up interviews (1 hour)	Direct observation during ward rounds and post-ward round EHR usage (4 hours), semistructured follow-up interviews (1 hour)
**Data analysis**	Transcribing and subsequent bottom-up coding of audiovisual recordings in ATLAS.ti	Transcribing and subsequent bottom-up coding of audiovisual recordings in ATLAS.ti	Transcribing and subsequent bottom-up coding of audiovisual recordings in ATLAS.ti

### Phase I: Data Collection

#### Data Collection Methods

Workarounds have been identified, analyzed, and described in various health care contexts, and in different ways; these include observations [32], interviews [20,31], focus groups [30], questionnaire surveys [33], document analyses, time and motion measures, information systems data analyses, and predominantly a combination of the foregoing methods [34]. In our study, we will apply nonparticipant direct observation in combination with semistructured post-observation interviews for two main reasons. First, workarounds must be observed *in vivo,* while work practices and EHR use by health care professionals unfold *in situ* [38]. Second, and related to the former, workarounds tend to be invisible: EHR users may not have any interest in making their workarounds explicit in interviews or surveys. In fact, if users reveal their workarounds, they could be held accountable for not complying with guidelines of system use [38].

Direct observations will be complemented with a follow-up semistructured interview with each observed health care provider. Although we will make use of an interview protocol with predefined questions that are of particular importance to maintain coherency across the cases being studied [39], not all questions will necessarily be asked in a fixed order (or asked at all). New questions may be formulated during the interviews to gather more in-depth data related to the subject being discussed, or an issue being raised by the interviewee. The questions will primarily be related to the direct observations in which the interviewee is the main actor. We therefore maintain a more flexible approach by actively probing and listening to the interviewee, known as an *open-ended interview* [40]. This approach facilitates the use of a standardized list of questions, thereby enhancing internal validity and reliability, while retaining a degree of flexibility to adapt to situational interests ad hoc [39]. Each physician, nurse, and clerk will be observed and interviewed independently. If a potentially preventable medical error occurs while observing or interviewing a participant (and only if the workaround poses a serious and direct threat, to maintain our nonparticipant approach to observation), the participant will first be asked about the reason for their workaround, and later be informed of any potential preventable medical error(s) that may result from the workaround. In addition, although an estimation of the number of physicians, nurses, and clerks to be observed and interviewed is provided in Table 1, observations and interviews will continue until the research team agrees that data saturation is achieved.

**Figure 3:**
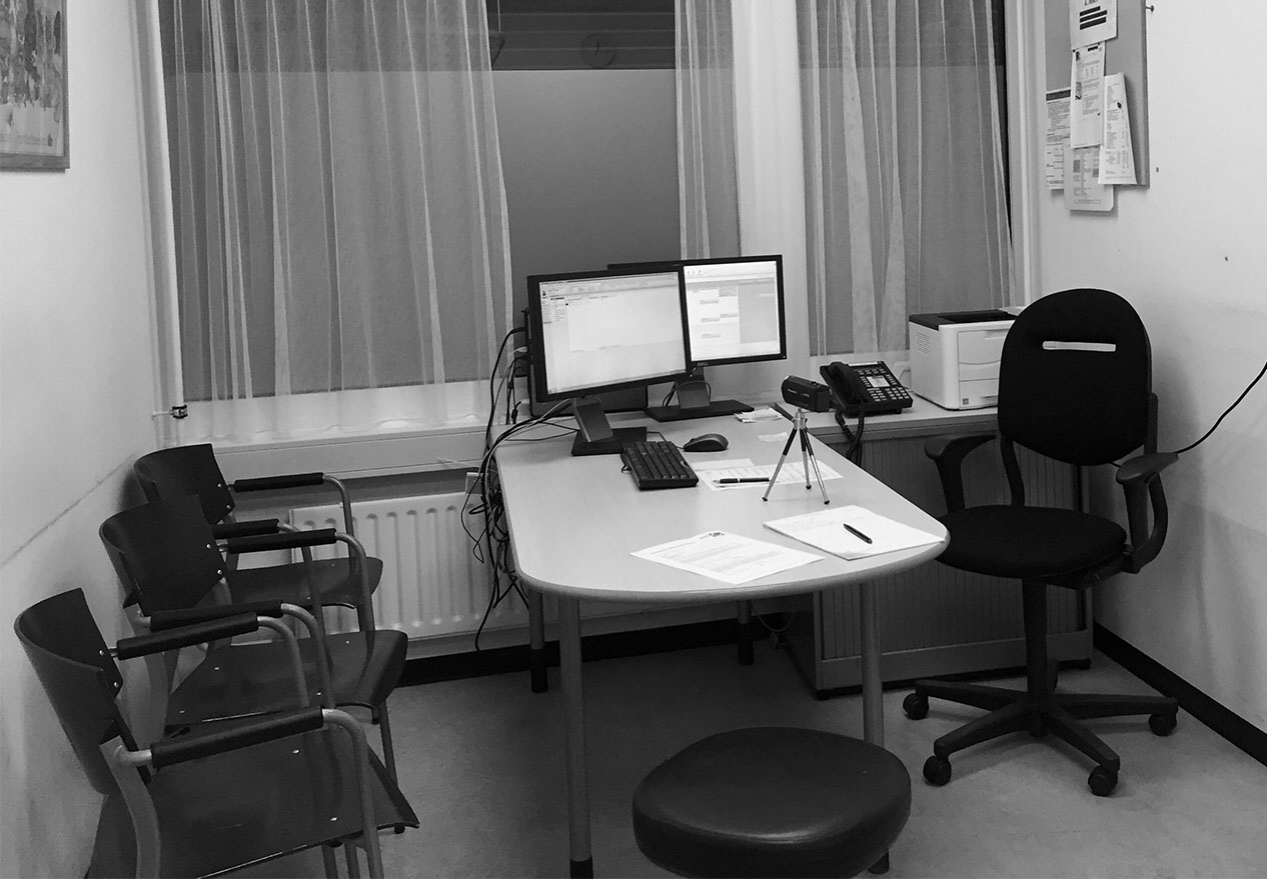
Example of a data collection setup in an outpatient consultation room.

All direct observations and interviews will be captured by means of a small audiovisual camera positioned at a designated, static location (see Figure 3 for an example setup). This procedure allows us to gather raw data from health care professionals and clerks using the system from which workarounds can be identified, to pinpoint moments in the processes when workarounds occur, and conduct follow-up analyses to gain deeper insights into how and why the workaround occurred. To mitigate the Hawthorne effect during the observations and audiovisual recordings, we will clearly communicate to the participants *what is in it for them*. It will be made clear that participants have no reason to use their EHR in any different way than they normally would. First, we will explain that participating in the research project is an opportunity to improve the EHR and thereby reduce potentially negative impacts on patient safety, effectiveness, and efficiency of care. Moreover, we will stress that we will be observing the EHR rather than the participant him/herself. Second, we will clearly communicate to participants that all data gathered will be anonymous, cannot be traced back to them, and will not be shared with anyone but the research team. This approach will reassure participants that they can use their EHR as they normally would, without fear of potentially being reprimanded or rebuked afterwards. Third, the audiovisual camera will be permanently and unobtrusively installed for the duration of the observation, and does not require frequent maintenance or recalibration. Finally, observers will be positioned at a *safe distance* from the clinician using the EHR [41].

The audiovisual recordings will be imported into a software application named ATLAS.ti and will be subject to further processing after this first research phase. All physicians, nurses, clerks, and patients will be asked for informed consent before any audiovisual recording takes place. The study has been proposed to, and discussed with, the chief of medical staff and the director of operations. We gained approval and support from these parties to proceed with the study, and no institutional review board approval was required. To protect patients’ health information, all audiovisual recordings will be stored on an encrypted hard drive set to erase itself after a series of incorrect password entries, and subject to editing, during which any patient names or contact details will be blurred or blanked out.

#### Three Distinct Processes

To study the EHR from a broader perspective within the university hospital, data will be gathered within three pediatric specialties: hematology, immunology, and infectious diseases. These specialties use the same EHR, of which the *look and feel* is identical, but may use additional functionalities tailored to each specialty. Within each specialty, providers will be observed while using the system in three distinct processes: the preparation of outpatient consultation, providing outpatient consultation, and providing inpatient consultation. We will deliberately analyze multiple distinct processes to create variation with regard to physical environment in which the EHR is used, tasks performed, and outcomes produced (see Figure 1). We expect to find different types of context-dependent workarounds in each of these processes. The preparation and data collection procedures per process are explained in the following subsections.

##### Process 1: Preparation of Outpatient Consultation

The first process concerns health care professionals preparing outpatient visits while using the EHR in their private offices. When preparing outpatient consultations an EHR user (eg, physician or nurse) primarily relies on the availability, retrievability, and quality of the data stored in the EHR by colleagues and the user him/herself, as important clinical information is often hidden in a *sea of data* [42]. Patients will not be present in this setting and the system user will not have direct interactions with other colleagues on site, so opportunistic questions may be asked during direct observations of users interacting with the EHR. Furthermore, users will be asked to explain how they use and navigate the EHR to provide richer insight into their actions. After the observed session, a semistructured interview will be conducted with the observed user to enrich the initial observations.

##### Process 2: Actual Outpatient Consultation

The second process of an outpatient consultation concerns obtaining the medical history from patients, conducting a physical examination, and ordering laboratory tests or medication while a health care professional uses the EHR in a designated examination room. Since these activities are regularly carried out in a limited time frame per patient, the ratio between provider-system and provider-patient interaction demands careful balancing [43-45]. Furthermore, unexpected complexity of tasks at hand may dictate workflow or EHR-functionality issues, may cause *no correct path* situations when a desired option does not exist in the system workflow, or spark data organization issues when a user would prefer a different overview of existing data (eg, historical patient data) [22]. Despite such issues, the user must still devise a way to accomplish his or her intentions within the given time frame; this likely gives rise to workarounds. Similar to the first process to be studied, a semistructured interview will be conducted after the observed session of the user interacting with the EHR in the presence of the patient.

##### Process 3: Actual Inpatient Consultation

The third and final setting concerns health care professionals using the EHR after having made inpatient ward rounds. These rounds concern regular daily reviews and consultation of hospitalized patients with regard to their medical condition, medication, and progress. Subsequently, the EHR is used to change drug prescriptions, order blood or other laboratory tests, and document the patient visit. The interface of the EHR differs from the interface shown to users in the first and second processes, thereby providing unique insights into usability-related workarounds. Similar to the first and second processes to be studied, a semistructured interview will be conducted after the observed session of the user interacting with the EHR.

### Phase II: Data Preparation

#### Transcribing

After the data collection phase, we will transcribe the audiovisual recordings of the direct observations and interviews. We will purposefully transcribe the recordings ourselves, as this will aid in data interpretation by developing affinity with the transcriptions. Each audiovisual recording of the observations and interviews will be transcribed in a separate Microsoft Word document. These files, including the original audiovisual recordings, will be imported into ATLAS.ti as *primary documents* within a hermeneutic unit. Within these primary documents, *quotations* will be created for selected text sections or video frames possibly related to a workaround resulting from EHR usage. Quotations are independent objects without any codes assigned to them and can be regarded as bookmarks within the data set. After all transcriptions have been processed, the research team will jointly review each transcription and quotation to determine whether (1) the quotation indeed relates to a workaround of the EHR, (2) there is consistency among the quotations in terms of the range (eg, selected string length or number of video frames) of the selected data, (3) minimal discrepancies exist between the audiovisual fragments and transcribed text, and (4) to ensure no relevant sections of data were overlooked.

#### Bottom-Up Versus Top-Down Coding

Two approaches to coding have been considered while designing this study: *top-down* and *bottom-up*. Top-down coding would require the configuration of a set of predetermined codes from existing literature on EHR workarounds [22,28,34]. We would then simply match our data against the predetermined codes and develop new codes if a quotation would not fit in the existing classification of codes. In contrast, using bottom-up coding, we would develop the codes ourselves with the naming of codes constantly being tentative and subject to change. As more data is analyzed over time, the tentativeness of the coding taxonomy would eventually develop itself into a set of codes that fit the data well [46]. Despite being more time-demanding, we will use a bottom-up approach, as this will allow us to generate potentially new types of categories that may not emerge from a top-down approach, due to potential analytical bias (ie, forcing data into predetermined categories).

#### Provisional Coding Taxonomy Development

In line with a bottom-up approach, a provisional coding taxonomy will be developed by the lead researcher based on impressions and notes taken during each observation and interview, before coding of the transcriptions commences. This provisional coding taxonomy provides the coding team with a birds-eye overview of what has been witnessed during the EHR sessions with users in each of the three processes. This process will generate a temporary list of codes to be assigned to the data, to prevent each coder from developing a unique list of codes, and to ensure that coders use the same names for sections of data when their interpretations of the transcriptions are identical.

#### Coder Training

To establish common ground among members of the coding team before coding the transcriptions, one or multiple plenary educational sessions will be organized in which the team will be instructed on the EHR, the coding scheme, the contents of the provisional coding taxonomy, the meaning of each code, and the basics of coding in ATLAS.ti. To achieve a sufficient level of consistency and quality among coders, they will be asked to code the same copy of a random interview transcription using the provisional coding taxonomy before the actual process of coding the transcriptions starts. The copies will then be merged in ATLAS.ti to create a single analyzable file that contains all actions performed by all coders. Results will then be compared and any discrepancies or ambiguities will be discussed. If the coding scheme turns out to be ambiguous, the lead researcher will adjust the taxonomy and coding responses will be recalibrated.

### Phase III: Identifying Workarounds

#### Open Coding

After the provisional coding taxonomy is finalized, the coding team will begin open coding. Initially, two coders will independently code five similar randomly chosen transcriptions using the provisional coding taxonomy. One or multiple codes may be assigned to each quotation. When data do not fit into codes of the provisional taxonomy, new codes may be proposed by the coders. Coders may likewise propose alternative ways of labeling the codes. The research team will then come together and compare the results of the coders. Any discrepancies related to the codes assigned for the same unit of text or video stills will be resolved through discussion. The provisional coding taxonomy will be adjusted accordingly by the lead researcher and in collaboration with the coders, if deemed necessary. Whenever the coding taxonomy is altered throughout the research project, the transcriptions that were already processed will be reviewed again to determine whether all quotations assigned to a code still match the revised coding taxonomy. The same holds true if a code has been broken into multiple codes, or multiple codes have been merged into a single one.

We expect the tentativeness of the coding taxonomy to develop itself into a set of codes that fit the data well, after this initial round of coding. Most of the remaining transcriptions will be independently coded by the coding team. An independent reviewer will review the coded transcriptions on a regular basis and signal the research team if inconsistencies are noticed (eg, continuously using an inappropriate code for quotations with similar semantics). The research team will then resolve the inconsistencies to ensure that the predefined codes are used by all coders in the same way.

#### Calculating Interrater Reliability and Interrater Agreement

When all transcriptions have been coded and validated by the research team, a random sample of identical transcriptions that have been independently coded by at least two coders will be merged in ATLAS.ti. This process will create a single analyzable file containing all actions performed by all coders. Within this file, interrater reliability and interrater agreement of codes assigned to transcriptions will be calculated. We aim to do this for 30% of the transcriptions (usually 10-20% [47]). Interrater reliability will tell us whether there is consistency among the coding team with regard to selecting the same codes for the same unit of analysis (ie, quotation) while coding in isolation. Interrater agreement will tell us the extent to which coders are able to reconcile through discussion (and mediation by the independent reviewer in case the coders fail to reach consensus) if coding discrepancies arise for the same unit of analysis [48].

#### Tabulation

Finally, the number of quotations associated with each code will be tabulated to provide insights into which codes are more prevalent, both overall and within each of the three different health care settings. A high number of quotations associated with a given code may prompt further investigation during follow-up interviews and provide clues as to why the given code occurs more often than others.

### Phase IV: Determining the Scope of Workarounds

The fourth phase aims to analyze the identified workarounds regarding their scope. As previously mentioned, each workaround will be related to its impact on the patient, the health care professional, the overall organization, or a combination thereof; this is in accordance with the *outcome* part of a process influenced by a workaround, as shown in Figure 1. Clues about which stakeholders are impacted by each workaround will primarily be gathered from the semistructured follow-up interviews. We will specifically look for responses that provide insights into the conditions that caused or influenced the workaround, the context in which it appeared, and the high-level consequences of the workaround. Any part of a response providing such insight will be stored in a separate file unique to each workaround. These files will be subject to further analyses in the following research phases.

### Phase V: Determining the Consequences of EHR Workarounds

The fifth phase involves determining the consequences of each identified workaround. We have been inspired by the approach of Ash et al [49] who present a thematic hierarchical network model of consequences of CPOEs that helps in building understanding of CPOE consequences. CPOE is built upon the diffusion of innovations theory [50] and distinguishes between three classifications of outcomes: anticipated versus unanticipated, desirable versus undesirable, and direct versus indirect. We believe this model fits well with our aim of determining the impact of an EHR workaround by classifying its consequences. For our study, we are limiting ourselves to unanticipated consequences and whether each of the identified consequences is regarded as desirable or undesirable (Figure 2). We will purposefully exclude anticipated consequences of EHR workarounds from our analyses, as the focus of this study is on workarounds that are inherently suboptimal and their consequences (by definition) are not anticipated by EHR designers. Furthermore, we do not distinguish between direct versus indirect consequences of EHR workarounds since consequences of workarounds elicited by EHR use may manifest themselves in the far future rather than the near future. The following subsections define unanticipated desirable versus undesirable consequences in an EHR workaround context.

#### Desirable Unanticipated Consequences of EHR Workarounds

An unanticipated consequence is a consequence of an EHR workaround that has not been foreseen in advance [49]. An unanticipated consequence can be either desirable or undesirable. Desirable unanticipated consequences are unforeseen consequences that turn out to have a favorable impact on an individual or the social system in which the EHR workaround occurred; these consequences can be described as *serendipity*. In the words of Ash et al [49], these consequences can be regarded as, “happy surprises”. Examples include increased collaboration and learning from alert messages, or ordering a wrong drug purposefully to trigger the alert system to suggest the right one.

#### Undesirable Unanticipated Consequences of EHR Workarounds

Undesirable unanticipated consequences are unforeseen consequences that turn out to have an unfavorable impact on an individual or the social system in which the EHR workaround occurred; these consequences can be termed *unintended consequences*. Examples include health care professionals suffering from *alert fatigue* due to an overload of alerts generated by the EHR with low specificity [19,25], paper persistence [25], deteriorated communication and cooperation among health care professionals [51-53], workflow issues [25], difficulties in finding information in the system [24], never-ending system demands [25], or a human-computer interface that is unsuitable for a highly interruptive health care context [26].

### Phase VI: Determining the Possible Impact of EHR Workarounds

The final phase involves determining the possible impact of EHR workaround consequences. One or multiple sessions will be organized to convene all members of the research team, health care professionals, and clerks participating in the study. The impact of each workaround consequence will then be collectively analyzed from three perspectives: patient safety, effectiveness of care, and efficiency of care.

A comprehensive list of indicators to determine the impact of EHR workaround consequences regarding the three perspectives is, to our knowledge, nonexistent. We will therefore develop a list of indicators following a bottom-up approach. Based on this list of indicators and garnered insights, our final aim is to develop a model of EHR workaround consequences and their possible impact on patient safety, effectiveness, and efficiency of care that builds upon the CPOE consequences model developed by Ash et al [49]. A concise description of the three perspectives is provided below.

#### Patient Safety

Patient safety is a broad discipline that has garnered increasing attention since the 1990s and has become a cornerstone of delivering high-quality health care [54]. The Institute of Medicine defines patient safety as, “the prevention of harm to patients” [55]. To tailor this definition to our context, we define patient safety as any EHR-related incident that could possibly harm one or multiple patients receiving care.

EHRs are regarded as essential to improving patient safety [7]. However, recent evidence highlights substantial and often unanticipated patient risks resulting from the use of EHRs or workarounds [34,56-58]. The safe delivery of patient care can be jeopardized by bypassing security blocks (eg, working in *emergency mode* in nonemergency situations and thereby omitting security checks) [28], cloaking deficiencies (ie, devising workarounds rather than bringing problems to the attention of systems designers, causing problems to remain hidden) [59], and undermining standardization (eg, using an alternative way to accomplish a task, thereby not conforming to a system-enforced way of working that is designed to safeguard patient safety) [60]. Understanding how consequences of EHR workarounds could impact patient safety is therefore key when formulating design and redesign interventions for EHRs.

#### Effectiveness of Care

According to ISO 9241-11 (1998), effectiveness can be defined as the accuracy and completeness with which users achieve specified goals [61]. Workarounds often have a negative impact on the effectiveness of user-EHR interaction; specifically, they have been found to result in information on patient care (processes) or work protocols that are unstable, unavailable, or unreliable [27]. To achieve closer alignment between work context and EHR design, it will be important to understand the impacts that workaround consequences have on the accuracy and completeness of the goals that EHR users hope to achieve.

#### Efficiency of Care

According to ISO 9241-11 (1998), efficiency can be defined as resources expended in relation to the accuracy and completeness with which EHR users achieve goals [61]. As previously mentioned, the ratio between provider-EHR and provider-patient interaction demands careful balancing [43-45]. Recent evidence confirms that EHRs claim a significant portion of physicians' time and draw attention away from their direct interactions with patients, and from their personal lives [62,63]. Physicians may spend 3-4 hours on EHR tasks and desk work for every hour of direct clinical time spent with patients [63]. Similar to the effectiveness of care potentially being jeopardized by workarounds, the same holds true for efficiency of care, as work protocols enforced by EHRs that are unstable, unavailable, or unreliable are sources of inefficiency [27].

## Results

Data was collected using the described approach from January 2016 to March 2017. Data analysis is underway and is expected to be completed in May 2017. We aim to report the results of this study in a follow-up publication.

## Discussion

Health care providers resort to informal work practices known as *workarounds* to handle exceptions to normal workflow unintentionally imposed by EHRs. Although these workarounds may seem favorable at first sight, they are generally suboptimal and may jeopardize patient safety, effectiveness, and efficiency of care. Understanding why and how workarounds occur is pivotal to developing user-friendly EHRs, and to achieve greater alignment between work context and the EHR.

Research on the scope and impact of EHR usage-related workarounds on the overall patient care processes is currently limited. Insights into the consequences of EHR workarounds on patients, health care providers, and health care organizations provide guidance on how to address EHR-related safety, effectiveness, and efficiency concerns, and to optimize EHR designs.

Our study protocol, based on the SEIPS conceptual framework [35], the thematic hierarchical network model [49], and an ethnographic approach using a combination of direct observations, semistructured interviews, and qualitative coding techniques provides a grounded framework to explore EHR workarounds from a holistic and integral perspective. More specifically, workarounds emerging from EHR use in three different health care settings will be assessed on their scope (ie, patient-, professional- or organization-related), consequences (ie, desirable versus undesirable) and impact (on patient safety, patient care effectiveness, and efficiency). Insights from this study can inform the redesign of our EHR to further align with these work contexts and subsequently lead to better organization and safer provision of care.

In addition to reporting on identified workarounds to EHR usage in an academic hospital in multiple distinct processes and settings, our final aim is to develop a model of EHR workaround consequences and their impacts on patient care that builds upon the CPOE consequences model developed by Ash et al [49]. This model will benefit researchers and practitioners alike when analyzing EHR workarounds, and subsequently in their efforts to improve EHR design for optimal EHR usage in health care practice.

## Abbreviations

CPOE: Computerized Physician Order Entry
EHR: Electronic Health Record
SEIPS: Systems Engineering Initiative for Patient Safety
